# The basic reproductive ratio of Barbour’s two-host schistosomiasis model with seasonal fluctuations

**DOI:** 10.1186/s13071-017-1983-1

**Published:** 2017-01-25

**Authors:** Shu-Jing Gao, Hua-Hua Cao, Yu-Ying He, Yu-Jiang Liu, Xiang-Yu Zhang, Guo-Jing Yang, Xiao-Nong Zhou

**Affiliations:** 1 National Institute of Parasitic Diseases, Chinese Center for Disease Control and Prevention; Key Laboratory of Parasite and Vector Biology, MOH, WHO Collaborating Center for Tropical Diseases, Shanghai, 200025 China; 2 Key Laboratory of Jiangxi Province for Numerical Simulation and Emulation Techniques, Gannan Normal University, Ganzhou, 341000 China; 3 Jiangsu Institute of Parasitic Diseases, Key Laboratory on Control Technology for Parasitic Diseases, Ministry of Health, Wuxi, Jiangsu 214064 China; 4 Swiss Tropical and Public Health Institute, Basel, Switzerland

**Keywords:** Schistosomiasis japonica, Mathematical model, Seasonal fluctuation, Basic reproductive ratio, Parameter estimation, Barbour’s two-host model

## Abstract

**Background:**

Motivated by the first mathematical model for schistosomiasis proposed by Macdonald and Barbour’s classical schistosomiasis model tracking the dynamics of infected human population and infected snail hosts in a community, in our previous study, we incorporated seasonal fluctuations into Barbour’s model, but ignored the effect of bovine reservoir host in the transmission of schistosomiasis. Inspired by the findings from our previous work, the model was further improved by integrating two definitive hosts (human and bovine) and seasonal fluctuations, so as to understand the transmission dynamics of schistosomiasis japonica and evaluate the ongoing control measures in Liaonan village, Xingzi County, Jiangxi Province.

**Methods:**

The basic reproductive ratio *R*_0_ and its computation formulae were derived by using the operator theory in functional analysis and the monodromy matrix theory. The mathematical methods for global dynamics of periodic systems were used in order to show that *R*_0_ serves as a threshold value that determines whether there was disease outbreak or not. The parameter fitting and the ratio calculation were performed with surveillance data obtained from the village of Liaonan using numerical simulation. Sensitivity analysis was carried out in order to understand the impact of *R*_0_ on seasonal fluctuations and snail host control. The modified basic reproductive ratios were compared with known results to illustrate the infection risk.

**Results:**

The Barbour’s two-host model with seasonal fluctuations was proposed. The implicit expression of *R*_0_ for the model was given by the spectral radius of next infection operator. The *R*_0_ *s* for the model ranged between 1.030 and 1.097 from 2003 to 2010 in the village of Liaonan, Xingzi County, China, with 1.097 recorded as the maximum value in 2005 but declined dramatically afterwards. In addition, we proved that the disease goes into extinction when *R*_0_ is less than one and persists when *R*_0_ is greater than one. Comparisons of the different improved models were also made.

**Conclusions:**

Based on the mechanism and characteristics of schistosomiasis transmission, Barbour’s model was improved by considering seasonality. The implicit formula of *R*_0_ for the model and its calculation were given. Theoretical results showed that *R*_0_ gave a sharp threshold that determines whether the disease dies out or not. Simulations concluded that: (i) ignoring seasonality would overestimate the transmission risk of schistosomiasis, and (ii) mollusiciding is an effective control measure to curtail schistosomiasis transmission in Xingzi County when the removal rate of infected snails is small.

**Electronic supplementary material:**

The online version of this article (doi:10.1186/s13071-017-1983-1) contains supplementary material, which is available to authorized users.

## Background

Schistosomiasis japonica, a parasitic disease caused by *Schistosoma japonicum*, has been in existence in the People’s Republic of China (P. R. China) for over 2000 years with considerable public-health and economic significance [1, 2]. A large-scale national schistosomiasis control programme was initiated in the mid-1950s [2, 3], when China’s population was approximately 600 million. An estimated 11.8 million people were infected with *S. japonicum* [4]. Sustained control efforts have contributed significantly to the dramatic reduction of both transmission intensity and schistosomiasis distribution in China in the past six decades [5–9]. Recent data have shown that progress toward schistosomiasis elimination encountered difficulties and setback due to high re-infection rates, particularly in lake region of the endemic areas [10]. For instance, schistosomiasis re-emerged shortly after the termination of The World Bank Loan Project (WBLP) at the end of 2001 [1, 5, 11]. In order to achieve the goal of schistosomiasis elimination in P. R. China, Chinese government strengthened the national schistosomiasis control programme in 2004. This made schistosomiasis control top priority along with the list of other communicable diseases such as HIV/AIDS, tuberculosis, and hepatitis B in China [4, 12]. Moreover, a revised strategy to effectively control schistosomiasis by using integrated measures in the national control programme has been implemented since 2005 [11].

Over 40 different species of wild and domestic animals have been identified as definitive hosts of *S. japonicum* [13]. Bovines are the major reservoirs for *S. japonicum* in the lake and marshland regions of southern China [14, 15]. A large number of schistosome-infected bovines are distributed in these regions and they excrete large quantities of *S. japonicum* eggs, the majority of which are deposited near or in the lake [14]. The daily faecal output from a water buffalo (~25 kg) has been estimated to be at least 100 times more than that (~250 g) from an individual human [16, 17]. It was reported that the overall prevalence of *S. japonicum* was 9.6 and 7.2% in water buffalo and cattle, respectively, in 1995 [18]. High prevalence of *S. japonicum* infection in bovine reservoir hosts is believed to be the major factor maintaining active transmission in certain areas. A cluster randomized intervention trial which was designed to compare the control (human treatment) and intervention (human and bovine treatment) in some villages concluded that the incidence of human *S. japonicum* infection is reduced with a decline in the infection rates of water buffaloes [19]. In order to remove bovines as a source of infection, several effective measures, including replacing cattle with farm machinery, isolating marshland, and prohibiting grazing in susceptible areas, have been implemented.

Mathematical modelling is a powerful tool to study the transmission dynamics of schistosomiasis [20]. It was first proposed by Macdonald [21]. Following this pioneering work, many mathematical models have been developed, all of which show great potential in aiding our understanding of the interplay of biology, transmission dynamics and control of schistosomiasis [22–27]. In 1996, Macdonald’s model was improved by Barbour [28]. It tracks dynamics of both infected human and snails in a community. Barbour’s model has played an important role in evaluating possible control strategies [29]. Traditionally, schistosomiasis models assume that all parameters are constant. A real world environment is obviously non-stationary, and should include seasonal variations in snail population. Infection rates vary seasonally due to natural factors (i.e. changes in moisture and temperature) and social factors (i.e. changes in contact rates). Therefore, it is more realistic to assume that the infection rates are periodic rather than constant. In our previous study [30], we constructed the Barbour’s single-host model with seasonal fluctuations (BSHSF model) and calculated the basic reproductive ratio to assess the effect of integrated control measures against schistosomiasis in Liaonan village, Xingzi County, Jiangxi Province. However, the impact of the bovine reservoir host on the transmission of schistosomiasis should not be ignored.

This study aims to further modify the Barbour’s two-host model with seasonal fluctuations (BTHSF model) and give an implicit expression of the basic reproductive ratio of schistosomiasis and computation method. Also, we will further establish some indexes that predict prevalence variation characteristics of schistosomiasis, and evaluate the prevention and control strategies for schistosomiasis.

## Methods

### Mathematical formulation

The Barbour’s single-host model with seasonal fluctuations (BSHSF model) in Gao et al. [30] was given by the following equations:

1 $$ \left\{\begin{array}{l}\frac{dP}{dt}= a(t)\Delta y\left(1- P\right)- gP,\hfill \\ {}\frac{dy}{dt}= b(t)\left(\frac{\sum }{\Delta}\right) P\left(1- y\right)-\mu y,\hfill \end{array}\right. $$

All variables of Eq. (1) are described in Table 1. The infection rate *a*(*t*) represents the rate at which a single definitive host becomes infected at unit density of infected snails at time *t*. The infection rate *b*(*t*) is the rate at which snails become infected at time *t*. The two infection rates can be calculated as follows (see [28]):

$$ \begin{array}{cc}\kern1em  a={a}_1{a}_2{a}_3,\kern1em & \kern1em  b={\beta}_1{\beta}_2{\beta}_3{\beta}_4,\kern1em \end{array} $$

**Table 1:** Interpretation of the model (1)

Parameter	Interpretation
*a*(*t*)	the rate of incidence for a single definitive host at unit density of infected snails at time *t*
*b*(*t*)	the rate at which an infected definitive host causes snail infections at time *t*
*g*	the recovery rate for definitive host infections
Δ	the density of snails
Σ	the density of definitive hosts
*μ*	per capita removal rate of infected snails

*Abbreviations*: *P*, the prevalence of infection in the definitive host population; *y*, the proportion of infected snails

Here, α_1_ denotes the rate at which a host has contact with contaminated water per unit time, α_2_ denotes the density of cercariae, α_3_ denotes the probability that an encounter with cercaria leads to the establishment of an adult parasite, *β*_1_ is the rate of egg-laying, *β*_2_ is the probability of an egg developing into a miracidium, *β*_3_ is the probability that a miracidium penetrates a snail, *β*_4_ is the probability that miracidial penetration into an uninfected snail develops into infection.

Seasonality was taken into consideration by assuming that the infection rates *a* and *b* in model (1) were time-varying and periodic. *a*(*t*), *b*(*t*) were taken as the absolute value of sine functions with 365 days period, while the average values of functions *a*(*t*) and *b*(*t*) per year (365 days) were assumed to be equal to the values of parameters *a* and *b* in [31], respectively.

Furthermore, we considered the impacts of bovine reservoir host on the transmission of schistosomiasis. The BSHSF model (1) was modified and used to create the BTHSF model. The dynamics of the model are governed by the following differential equations:

2 $$ \left\{\begin{array}{l}\frac{d{ P}_1}{ d t}={a}_1(t)\Delta y\left(1-{p}_1\right)-{g}_1{P}_1,\hfill \\ {}\frac{d{ P}_2}{ d t}={a}_2(t)\Delta y\left(1-{p}_2\right)-{g}_2{P}_2\hfill \\ {}\frac{d y}{ d t}=\left(\frac{b_1(t){\sum}_1{P}_1+{b}_2(t){\sum}_2{P}_2}{\Delta}\right)\left(1- y\right)-\mu y.\hfill \end{array}\right. $$

All variables of Eq. (2) are described in Table 2, where *a*_1_(*t*) denotes the rate of incidence for one human host at unit density infected by one infectious snail per unit time (one day) at time *t*, *a*_2_(*t*) denotes the rate of incidence for one bovine host at unit density infected by one infectious snail per unit time (1 day) at time *t*, *b*_1_(*t*) is the rate of incidence for one susceptible snail at unit density infected by one infected human per unit time (1 day) at time *t*, *b*_2_(*t*) is the rate of incidence for one susceptible snail at unit density infected by one infected bovine per unit time (1 day) at time *t*. In this study, we assume that *b*_1_ is proportional to *b*_1_, i.e. *b*_2_ = *kb*_1_, where *k* is the coefficient ratio. According to Zhao et al. [32]:

$$ k=\frac{b_2}{b_1}=\frac{A_2\times {B}_2}{A_1\times {B}_1}, $$

where *A*_*i*_ (*i* = 1,2) denote faeces output per human and bovine, *B*_*i*_ (*i* = 1,2) denote the epg of human and bovine, respectively, here epg is the number of eggs per gram of faeces. The number of eggs per bovine per day is more than 10^5^ [33], and the number of eggs per human per day is about ~2800–3000 [34]. Furthermore, many effective measures, such as improved sanitation and health education are implemented to control the spread of schistosomiasis among human beings. In this paper, we choose *k* =50. We also take the infection rates as:

$$ \left\{\begin{array}{c}\hfill {a}_1(t)={a}_{10}\Big| \sin \pi t/365\Big|,\hfill \\ {}\hfill {a}_2(t)={a}_{20}\Big| \sin \pi t/365\Big|,\hfill \\ {}\hfill {b}_1(t)={b}_{10}\Big| \sin \pi t/365\Big|,\hfill \\ {}\hfill {b}_2(t)={a}_{20}\Big| \sin \pi t/365\Big|.\hfill \end{array}\right. $$

**Table 2:** Interpretation of the model (2)

Parameter	Interpretation
*a*_1_(*t*)	the infection rate from snail to human at time *t*
*a*_2_(*t*)	the infection rate from snail to bovine at time *t*
*b*_1_(*t*)	the infection rate from human to snail at time *t*
*b*_2_(*t*)	the infection rate from bovine to snail at time *t*
*g*_1_	the recovery rate for human host infections
*g*_2_	the recovery rate for bovine host infections
Δ	the density of snails
Σ_1_	the density of human hosts
Σ_2_	the density of bovine hosts
*μ*	per capita removal rate of infected snails

*Abbreviations*: *P*_1_, prevalence of infection in the human host population; *P*_2_, the prevalence of infection in the bovine host population; *y*, the proportion of infected snails

Since infection rates *a*_*i*_ and *b*_*i*_ (*i* = 1,2) denote the average values of *a*_*i*_(*t*) and *b*_*i*_(*t*) per year, respectively, we have

$$ \begin{array}{cccc}\hfill {a}_1=\frac{1}{365}{\displaystyle \underset{0}{\overset{365}{\int }}{a}_1(t) dt},\hfill & \hfill {a}_2=\frac{1}{365}{\displaystyle \underset{0}{\overset{365}{\int }}{a}_2(t) dt},\hfill & \hfill \mathrm{and}\hfill & \hfill {b}_1=\frac{1}{365}{\displaystyle \underset{0}{\overset{365}{\int }}{b}_1(t) dt}.\hfill \end{array} $$

Thus we can derive $$ \begin{array}{cccc}\hfill {a}_{10}=\frac{\pi}{2}{a}_1,\hfill & \hfill {a}_{20}=\frac{\pi}{2}{a}_2,\hfill & \hfill {b}_{10}=\frac{\pi}{2}{b}_1,\hfill & \hfill {b}_{20}=\frac{\pi}{2} k{b}_1\hfill \end{array} $$ and

$$ \left\{\begin{array}{c}\hfill {a}_1(t)=\frac{\pi}{2}{a}_1\Big| \sin \pi t/365\Big|,\hfill \\ {}\hfill {a}_2(t)=\frac{\pi}{2}{a}_2\Big| \sin \pi t/365\Big|,\hfill \\ {}\hfill {b}_1(t)=\frac{\pi}{2}{b}_1\Big| \sin \pi t/365\Big|,\hfill \\ {}\hfill {b}_2(t)=\frac{\pi}{2} k{b}_2\Big| \sin \pi t/365\Big|.\hfill \end{array}\right. $$

### The basic reproductive ratio (*R*_0_)

For epidemiological models, the basic reproductive ratio, which is often introduced as a threshold parameter, is defined as the expected number of secondary infections produced by a single infective individual in a completely susceptible population during its entire infectious period [35]. *R*_0_ has often been used to predict the trend of the disease transmission and also to assess the effects of control measures. Here, we give the implicit formula *R*_0_ of model (2). It is expected that the infection will persist if *R*_0_ is greater than one. As *R*_0_ increases, the spreading rate of the disease also increases. This implies that more intervention efforts or improved control measures should be implemented. We have also shown that *R*_0_ is a sharp threshold value which determines whether the disease dies out or not in Additional file 1: Appendix B. That is, if *R*_0_ < 1, then the disease will die out, whereas if *R*_0_ > 1, the disease will be endemic.

With reference to Bacaër [36], a biologically meaningful threshold value *R*_0_ of BTHSF model (2) was derived by using operator theory in functional analysis and the monodromy matrix of linear periodic system theory. The numerical computation of the basic reproductive ratio was carried out using the mathematical programming language MATLAB 7.1. Also, sensitivity analysis was carried out in order to assess the impact of seasonal fluctuations on *R*_0_

### Study area and estimation of model parameters

Liaonan village, Xingzi County, Jiangxi Province was selected as the study area. The basic reproductive ratio was calculated based on the parameters in model (2). Determination of accurate model parameters was the priority when this threshold was applied. The details of the technique used in estimating the parameters have been described in our previous study [30]. In this paper, part of the parameters in model (2) were determined according to their biological significance. In addition, the remaining parameters were estimated based on the annual report surveillance data of Xingzi County from 2003 to 2010. The meanings of parameters *g*_*i*_, *Σ*_*i*_(*i* = 1,2), *μ*, *ω* are consistent with those reported in [30] (see Tables 1 and 2).

The parameters are stated as follows; g_1_ = 0.00093 per day, *μ* = 0.0055 per day, and g_2_ = 0.00183 per day (the data are given by Wu [31]). The density of snails, Δ(/*m* ^2^), and the density of definitive hosts, Σ_1_, Σ_2_(/m^2^), were estimated from annual report surveillance data (see Table 3).

**Table 3:** The data for Δ, Σ_1_, Σ_2_, ${\overline{P}}_1$, ${\overline{P}}_2$ and $\overline{y}$ from annual report data in the village of Liaonan, Xingzi County, Jiangxi Province, China

Parameter	2003	2004	2005	2006	2007	2008	2009	2010
Δ	27.997	33.9230	91.4602	133.5238	41.0615	17.4960	11.3121	2.4183
Σ_1_	0.0300	0.0199	0.0475	0.0167	0.0309	0.0178	0.0309	0.0176
Σ_2_	0.0013	0.0007	0.0020	0.0006	0.0010	0.0006	0.0013	0.0008
${\overline{P}}_1$	0.0671	0.0533	0.0631	0.0454	0.0454	0.0454	0.04530	0.0457
${\overline{P}}_2$	0.0283	0.1379	0.1756	0.1144	0.1084	0.0676	0.0639	0.0466
$\overline{y}$	0.0002	0.00005	0.0001	0.0001	0.0002	0.0005	0.0008	0.0015

It is assumed that the coefficients of model (2) are constant. By setting the right-hand side of equations in model (2) to zero, the steady-state (equilibrium) values for the prevalence of infection in humans, bovines and snails were obtained [28]

3 $$ \frac{1}{1-\overline{y}}=\frac{t_{MS}^{(1)}}{\overline{y}+1/{t}_{SM}^{(1)}}+\frac{t_{MS}^{(2)}}{\overline{y}+1/{t}_{SM}^{(2)}}, $$

4 $$ {\overline{P}}_i=\frac{\overline{y}}{\overline{y}+1/{t}_{SM}^{(i)}}, $$

where *t*_*MS*_^(*i*)^ = *b*_*i*_∑_*i*_/(*μΔ*) and *t*_*SM*_^(*i*)^ = *a*_*i*_ *Δ*/*g*_i_(*i* = 1, 2) are the transmission factors for definitive host *i*. By solving (3) and (4), we obtain the prevalence of infection in humans, bovines and snails from 2003 to 2010.

## Results

### Calculation of *R*_0_

Following the results of Bacaër [36] and Wang & Zhao [37], we show that *R*_0_ is equal to the value of *λ*, where *λ* is the positive root of the equation

5 $$ \rho \left( W\left(365,0,\lambda \right)\right)=1, $$

Here

$$ \begin{array}{cc}\hfill W\left(365,0,\lambda \right)= \exp \left[{\displaystyle {\int}_0^{365}\left(\begin{array}{ccc}\hfill \begin{array}{c}\hfill -{g}_1\hfill \\ {}\hfill 0\hfill \\ {}\hfill {b}_1(t){\sum}_1/\left(\lambda \Delta \right)\hfill \end{array}\hfill & \hfill \begin{array}{c}\hfill 0\hfill \\ {}\hfill -{g}_2\hfill \\ {}\hfill {b}_2(t){\sum}_2/\left(\lambda \Delta \right)\hfill \end{array}\hfill & \hfill \begin{array}{c}\hfill {a}_1(t)\Delta /\lambda \hfill \\ {}\hfill {a}_2(t)\Delta /\lambda \hfill \\ {}\hfill -\mu \hfill \end{array}\hfill \end{array}\right)} dt\right],\hfill & \hfill \forall \lambda >0\hfill \end{array}. $$

and *ρ*(*W*(365, 0, *λ*)) is the spectral radius of matrix *W*(365, 0, *λ*).

The mathematical details that were used to derive the expression for the basic reproductive ratio can be found in Additional file 1: Appendices A, B (Derivation of *R*_0_ and proof of main results).

### Numerical simulation

Based on the annual report data for Xingzi County from 2003 to 2010, the equilibrium values $\left({\overline{P}}_1,{\overline{P}}_2,\overline{y}\right)$ (prevalence of infection in human host, bovine reservoir host and snail) are shown in Table 3. In view of the parameters’ values given above (see Table 3), the infection rates *a*_1_, *a*_2_, *b*_1_ and *b*_2_ of BTH model was derived from Eqs. (3) and (4), and are shown in Table 4.

**Table 4:** The calculated values of composite parameters *a*_1_, *a*_2_, *b*_1_ and *b*_2_

Parameters	2003	2004	2005	2006	2007	2008	2009	2010
*a*_1_	0.0116	0.0309	0.0051	0.0024	0.0061	0.0050	0.0051	0.0120
*a*_2_	0.0093	0.1726	0.0320	0.0128	0.0305	0.0151	0.0144	0.0241
*b*_1_	0.0082	0.0015	0.0032	0.0260	0.0057	0.0171	0.0085	0.0075
*b*_2_	0.4084	0.0754	0.1606	1.2996	0.2871	0.8565	0.4268	0.3725

In Additional file 1: Appendix A, we illustrate that *R*_0_ is the positive root of the equation *ρ*(*W*(365, 0, *λ*)) = 1. Using the data in Tables 3 and 4, the values of *R*_0_ for BTHSF model were calculated and shown in Table 5. Let *R*_0_^′^ be the basic reproductive ratio for BSHSF model. It was calculated using established methods (see Table 6).

**Table 5:** The result of the basic reproductive ratio from annual report data in the village of Liaonan, Xingzi County, Jiangxi Province, China

The basic reproductive ratios	2003	2004	2005	2006	2007	2008	2009	2010
*R*_0_ (BTHSF model)	1.031	1.074	1.097	1.060	1.057	1.038	1.037	1.030
*ℜ*_0_ (BTH model)	1.052	1.142	1.192	1.114	1.107	1.066	1.064	1.050

**Table 6:** The result of the basic reproductive ratio from annual report data in the village of Liaonan, Xingzi County, Jiangxi Province, China

The basic reproductive ratios	2003	2004	2005	2006	2007	2008	2009	2010
*R*_0_ (BTHSF model)	1.031	1.074	1.097	1.060	1.057	1.038	1.037	1.030
*R*_0_^'^ (BSHSF model)	1.043	1.035	1.040	1.031	1.031	1.031	1.031	1.032

Barbour [28], proposed the following model without seasonality (BTH model):

$$ \left\{\begin{array}{l}\frac{d{ P}_1}{ d t}={a}_1\Delta y\left(1-{P}_1\right)-{g}_1{P}_1,\hfill \\ {}\frac{d{ P}_2}{ d t}={a}_2\Delta y\left(1-{P}_2\right)-{g}_2{P}_2,\hfill \\ {}\frac{d y}{ d t}=\left(\frac{b_1{\sum}_1{P}_1+{b}_2{\sum}_2{P}_2}{\Delta}\right)\left(1- y\right)-\mu y,\hfill \end{array}\right. $$

and obtained the basic reproductive ratio

$$ {R}_0=\frac{a_1{b}_1{\sum}_1}{g_1\mu}+\frac{a_2{b}_2{\sum}_2}{g_2\mu}. $$

Using the data in Tables 3 and 4, we obtain the values of *R*_0_ in the village of Liaonan from 2003 to 2010.

**Fig. 1:**
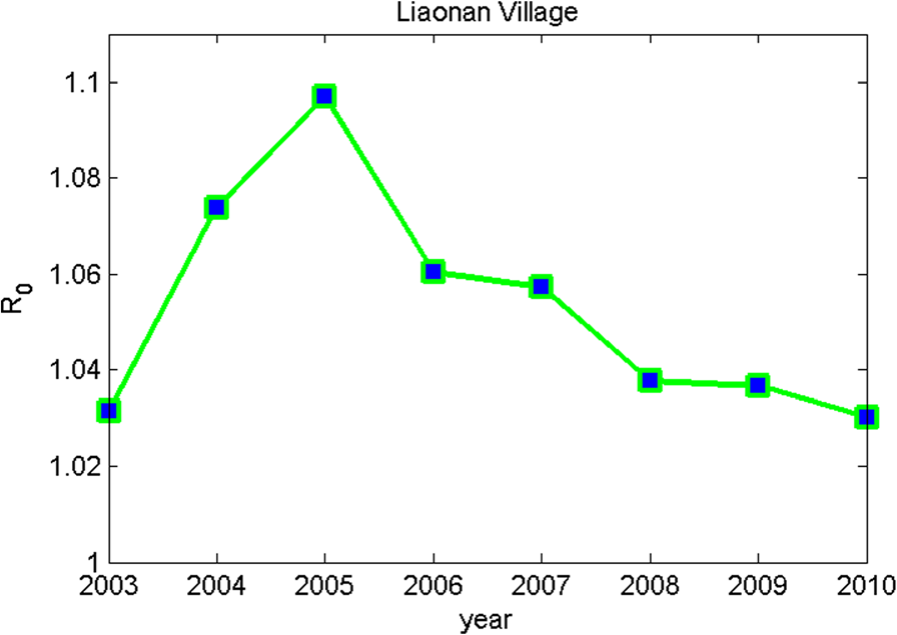
Changes in the basic reproductive ratio in Liaonan village from 2003 to 2010

*R*_0_ for BTHSF model is shown in Table 3, and it clearly shows that the values range between 1.030 and 1.097 from 2003 to 2010 in the village of Liaonan. Moreover, *R*_0_ for BTH model is always greater than that of BTHSF model at the same time. Furthermore, the variation tendency of *R*_0_ for BTHSF model is shown in Fig. 1.

The prevalence of two hosts and vector host has strong effect on *R*_0_ for BTHSF model. Except in 2003 and 2010, the values of *R*_0_ for BTHSF model are greater than those for BSHSF model in the other years (see Table 4). The prevalence of infected bovines in 2003 and 2010 are less than the other years (see Table 2). This phenomenon illustrates that if the prevalence ratio of infected bovine hosts and infected human hosts $\left({\overline{P}}_2/{\overline{P}}_1\right)$ is small, *R*_0_ for BTHSF model may be smaller than for BSHSF model. Moreover, we note that *R*_0_ and the prevalence of infection in bovines $\left({\overline{P}}_2\right)$ for BTHSF model are higher in 2004 and 2005 among the years under study as shown in (Tables 1 and 3).

### Sensitivity analysis

**Fig. 2:**
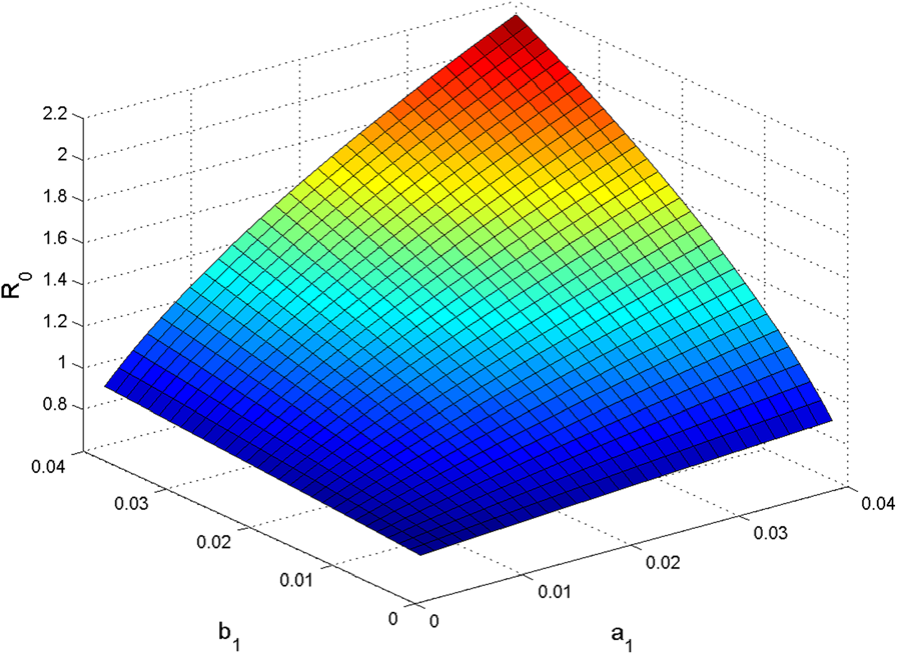
The relationship between *a*_1_, *b*_1_ and *R*_0_. The graph demonstrates the sensitivity of the basic reproductive ratio to the changes of composite parameters *a*_1_ and *b*_1_

**Fig. 3:**
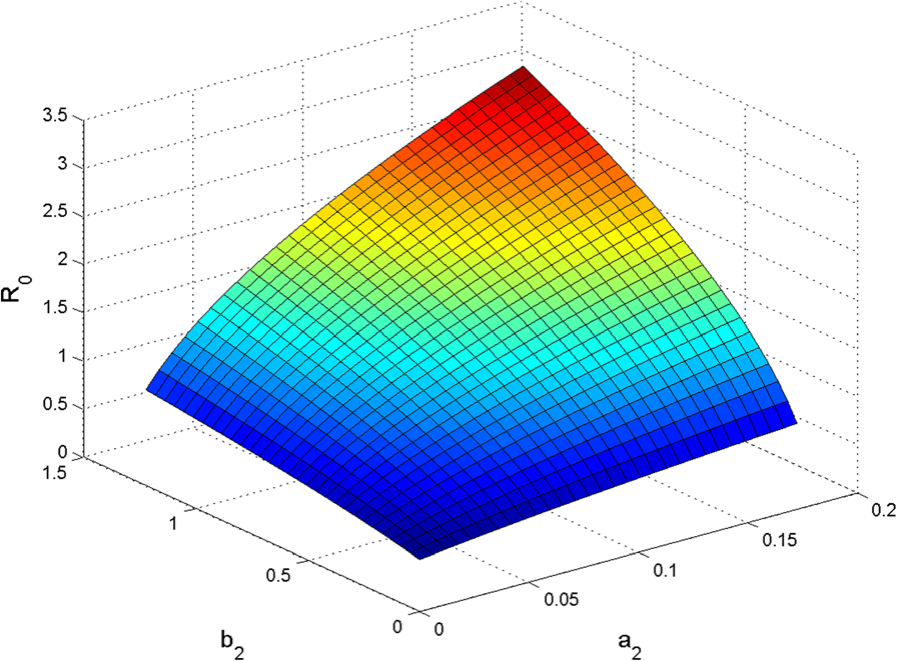
The relationship between *a*_2_, *b*_2_ and *R*_0_. The graph demonstrates the sensitivity of the basic reproductive ratio to the changes of composite parameters *a*_2_ and *b*_2_

**Fig. 4:**
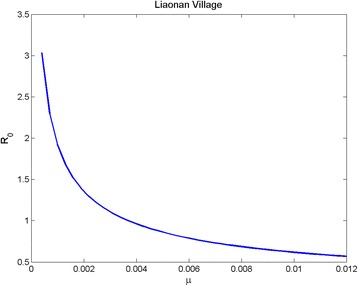
The relationship between *μ* and *R*_0_. The graph demonstrates the sensitivity of the basic reproductive ratio to the changes of parameter *μ*

In model (2), infection rates *a*_1_(*t*) and *b*_1_(*t*) (i = 1,2) were taken as sine functions of 365 days period, with the form ${a}_1(t)=\frac{\pi}{2}{a}_1\Big| \sin \pi t/365\Big|$ and ${b}_1(t)=\frac{\pi}{2}{b}_1\Big| \sin \pi t/365\Big|$. Thus, *πa*_*i*_/2 and *πb*_*i*_/2 reflect the amplitude of infection rates *a*_*i*_(*t*) and *b*_*i*_(*t*), respectively. We fix g_1_ = 0.00093, g_2_ = 0.00183, Δ = 91.4602, Σ_1_ = 0.0475, g_1_ = 0.00093, Σ_2_ = 0.0020, *μ* = 0.0055, *a*_2_ = 0.0426, *b*_2_ = 0.1224, and vary *a*_1_ and *b*_1_ in [0,0.04] in (5), with other parameters unchanged as above, by numerical simulations we obtain the curve of *R*_0_ with respect to *a*_1_ and *b*_1_ (see Fig. 2). It shows that the larger *a*_1_ and *b*_1_ are the higher *R*_0_ is. When *a*_1_ and *b*_1_ are near to 0, *R*_0_ is less than 1, which indicates that the control strategy is very effective and disease transmission will be interrupted. This implies that *R*_0_ is very sensitive to the changes in *a*_1_ and *b*_1_ when their values are higher than 0.03. Next, if we fix *a*_1_ = 0.0068, *b*_1_ = 0.0024, and let *a*_2_ and *b*_2_ vary. A graph indicating the relationship between *R*_0_ and *a*_2_ and *b*_2_ was obtained (Fig. 3). This graph shows that *R*_0_ increases with an increase in the amplitude of *a*_2_ and *b*_2_. Finally, as *μ* varies in [0,0.012] with other parameters unchanged as above, numerical simulations provide the relationship between *R*_0_ and *μ* (Fig. 4). Figure 4 shows that *R*_0_ is more sensitive to changes in *μ* when *μ* is less than 0.002.

## Discussion

This paper proposed Barbour’s two-host model with seasonality (BTHSF model), which is an improvement on the models BTH and BSHSF. Following the idea of Wang & Zhao [37], the implicit formula of *R*_0_ for BTHSF was given. By computation, the annual changing trend of *R*_0_ *s* in Liaonan village from 2003 to 2010 was given (see Fig. 1). It showed that *R*_0_ peaked in 2005 at 1.09 and declined dramatically until 2010. There are three possible factors responsible for this decline. First, in 2001, the World Bank Loan Project (WBLP) for schistosomiasis control (1992–2001) was terminated. This led to the resurgence of schistosomiasis transmission after 2001 [38, 39]. Secondly, the implementation of integrated control strategies with emphasis on infection source control commenced in 2005. Finally, the high prevalence of *S. japonicum* in bovines contributes to the high value of *R*_0_.

By numerical simulation, we realize that the basic reproductive number for Barbour’s model with constant infection rates is always higher than that of periodic infection rates. This illustrates that ignoring seasonal fluctuations would lead to overestimating schistosomiasis transmission risk.

Chemotherapy, health education, provision of water, sanitation and hygiene, bovine control and snail host control are the main measures for schistosomiasis control. We know that the implementation of chemotherapy strategy is beneficial to the recovery and well-being of patients and cattle. This would result in increasing the parameters *g*_1_ and *g*_2_ (recovery rates) in model (2). Accordingly, the implementation of control strategies, such as health education, provision of water, sanitation and hygiene, bovine control would lead to decrease in; the host’s contact rate with contaminated water, the probability that an encounter with cercariae results in the development of an adult parasite and the rate at which schistosome eggs are laid. Therefore, the infection rates (*a*_1_(t), *a*_2_(t), *b*_1_(t), *b*_2_(t)) in model (2) would decrease. More so, the implementation of snail control strategy will increase the removal rate of the snail (*μ*). Due to the implementation of preventive chemotherapy in Xingzi County, we assessed the sensitivity analysis of the average infection rates and removal rate. Numerical results have shown that *R*_0_ is a monotone increasing function of the four infection rates and a monotone decreasing function of the removal rate. This implies that as the infection rates increases or the removal rate decreases, *R*_0_ increases. Our results also indicate that mollusiciding is an effective means of controlling schistosomiasis transmission in Xingzi County when the removal rate of snails is small.

Finally, in line with the theoretical concept of this paper, we introduce several indexes that were explored extensively. Similar to the methods established in [28], *R*_0_ *s* are estimated from prevalence data and assumed to be at a steady equilibrium state, so they are always more than 1. In reality, *R*_0_ may be less than 1. However, we can study the transmission intensity by using the values of *R*_0_. For example, if we define the relative basic reproductive ratio as:

$$ {R}_i^j=\frac{R_{0 j}}{R_{0 i}} $$

where ${R}_{0_k}$ denotes the basic reproductive ratio of year *k*. If *R*_*i*_^*j*^ > 1, the transmission status of schistosomiasis in year *j* is more serious than year *i* and vice versa.

Next, we define the relative mean basic reproductive ratio as

$$ {\mathrm{\Re}}_m=\frac{R_{0_m}}{{\overline{R}}_0} $$

Here, ${\overline{R}}_0=\frac{1}{j- i+1}{\displaystyle \sum_{k= i}^j{R}_{0_k}\cdot {\mathrm{\Re}}_m>1}$ shows that the basic reproductive ratio in year *m* is greater than the average ratio from year *i* to *j*, and vice versa. Taking Liaonan village as the example, the relative basic reproductive ratio in 2005 and 2006 is defined as:

$$ {R}_{2005}^{2006}=\frac{R_{0_{2006}}}{R_{0_{2005}}}=\frac{1.12}{1.20}=0.9<1. $$

This illustrates that the epidemic trend in 2005 is relatively strong, compared to 2006. In addition,

$$ {\overline{R}}_0=\frac{1}{8}{\displaystyle \sum_{k=2003}^{2010}{R}_{0_k}=1.11,} $$

and

$$ {\mathrm{\Re}}_{2010}=\frac{R_{0_{2010}}}{{\overline{R}}_0}=\frac{1.06}{1.11}=0.96<1. $$

This indicates that the epidemic trends in 2010 are slower than the average level from 2003 to 2010.

Similarly, it is also able to measure the disease transmission situation between two different regions. Let ${R}_0^i,{\tilde{R}}_0^i$ denote the basic reproductive ratios of two different regions in year *i*, respectively. The index of the basic reproductive ratio is defined as:

$$ {\gamma}_i=\frac{R_0^i}{{\tilde{R}}_0^i}. $$

*γ*_*i*_ can be used to compare the effect of control and intervention between two regions at the same time. However, many factors such as heterogeneity of definitive host, drug resistance and optimal control method are yet to be considered in this mathematical model. These issues need to be addressed in the future studies.

## Conclusions

We modified and improved Barbour’s model to investigate the role of seasonal fluctuations in the spread of schistosomiasis. The formula for the basic reproductive number *R*_0_ with a single host was generalized with that of two hosts. Theoretical results showed that it is the basic reproductive ratio which is the threshold value that determines whether schistosomiasis should persist or not. The values of *R*_0_ in Liaonan village from 2003 to 2010 were calculated using the mathematical programming language MATLAB 7.1. The results from this study have shown that ignoring seasonality would overestimate the transmission risk of schistosomiasis, and mollusiciding would be the most effective control measure to curtail schistosomiasis transmission in Xingzi County. Also, the indexes established in this paper will lead to the re-evaluation of control strategies between different areas.

## Additional file

Additional file 1: Appendix A. Derivarion of R_0_. Appendix B. Proof of the main results. (PDF 67 kb)

## Abbreviations

BSH model: Barbour’s single-host model
BSHSF model: Barbour’s single-host model with seasonal fluctuations
BTH model: Barbour’s two-host model
BTHSF model: Barbour’s two-host model with seasonal fluctuations
epg: Eggs per gram
ERC: Institutional Ethics Review Committee
*R*_0_: The basic reproductive ratio
WBLP: The World Bank Loan Project
