# Supplementary material for: The basic reproductive ratio of Barbour’s two-host schistosomiasis model with seasonal fluctuations
Source: Parasit Vectors. 2017 Jan 25;10:42. doi: 10.1186/s13071-017-1983-1 (PMC5264280; doi:10.1186/s13071-017-1983-1)
Supplement: Additional file 1: — Appendix A. Derivarion of R0. Appendix B. Proof of the main results. (PDF 67 kb) [file 13071_2017_1983_MOESM1_ESM.pdf]

Appendix A: Derivation of R_0

In this part, we will describe the determination procedure for the implicit formula of the basic reproductive ratio of system (2.1) according to the general procedure established by Wang and Zhao [1]. It is easy to see that system (2.1) has exactly one disease-free solution $E_0 = (0, 0, 0)$.

Let $x = (P_1, P_2, \mathrm{y})^T$. Then model (2.1) can be written as

$$\frac{dx}{dt} = \mathscr{F}(t, x) - \mathscr{V}(t, x),$$

where

$$\mathscr{F}(t,x) = \begin{pmatrix} a_1(t)\Delta \mathrm{y}(1 - P_1) \\ a_2(t)\Delta \mathrm{y}(1 - P_2) \\ \left(\dfrac{b_1(t)\Sigma_1 P_1 + b_2(t)\Sigma_2 P_2}{\Delta}\right)(1 - \mathrm{y}) \end{pmatrix},$$

and

$$\mathscr{V}(t,x) = \begin{pmatrix} g_1 P_1 \\ g_2 P_2 \\ \mu \mathrm{y} \end{pmatrix}.$$

By the arguments similar to those in [2], it then follows that

$$D_x\mathscr{F}(t, E_0) = F(t) \text{ and } D_x\mathscr{V}(t, E_0) = V(t).$$

We can get

$$F(t) = \begin{pmatrix} 0 & 0 & a_1(t)\Delta \\ 0 & 0 & a_2(t)\Delta \\ \dfrac{b_1(t)\Sigma_1}{\Delta} & \dfrac{b_2(t)\Sigma_2}{\Delta} & 0 \end{pmatrix}, \quad V(t) = \begin{pmatrix} g_1 & 0 & 0) \\ 0 & g_2 & 0) \\ 0 & 0 & \mu \end{pmatrix}.$$

It is easy to see that $F(t)$ is non-negative, and $-V(t)$ is cooperative in the sense that the off-diagonal elements of $V(t)$ are non-negative. Let $\Phi_V(t)$ be the monodromy matrix of the linear ω-period system $\dfrac{dz}{dt} = V(t)z$ and $\rho(\Phi_V(\omega))$ be the spectral radius of $\Phi_V(\omega)$.

Let $Y(t, s)$ $(t \geq s)$ be a 3×3 matrix solution of the system

$$\frac{d}{dt}Y(t, s) = -V(t)Y(t, s), \quad \forall t \geq s, \quad \text{and } Y(s, s) = I,$$

where I is the 3×3 identity matrix. Thus, the monodromy matrix $\Phi_{-V}(t)$ of $\dfrac{dy}{dt} = -V(t)y$ is equal to $Y(t, 0), \quad t \geq 0$.

In view of the periodic environment, we assume that $\phi(s)$, ω-periodic in s, is the initial distribution of infectious individuals. Then $F(s)\phi(s)$ is the rate of new infections produced by

the infected individuals who were introduced at time s. Given $t \geq s$, then $Y(t,s)F(s)\phi(s)$ gives the distribution of those infected individuals who were newly infected at time s and remain in the infected compartments at time t. It follows that

$$\psi(t) = \int_{-\infty}^{t} Y(t,s)F(s)\phi(s)ds = \int_{0}^{\infty} Y(t,t-a)F(t-a)\phi(t-a)da$$

is the distribution of accumulative new infections at time t produced by all those infected individuals $\phi(s)$ introduced at time previous to t.

Let C_ω be the ordered Banach space of all ω-periodic functions from R to R^3, which is equipped with the maximum norm $\|\cdot\|$ and the positive cone $C_\omega := \{\phi \in C_\omega : \phi(t) \geq 0, \forall t \in R\}$. Then we can define a linear operator $L : C_\omega \to C_\omega$ by

$$(L\phi)(t) = \int_0^\infty Y(t,t-a)F(t-a)\phi(t-a)da, \ \forall\ t \in R,\ \phi \in C_\omega$$

where L is called the next infection operator. The basic reproduction rate of system (2.1) is defined as the spectral radius of L, i.e. $R_0 = \rho(L)$.

In order to characterize R_0, we consider the following linear ω-periodic equation

$$\frac{dw}{dt} = \left[-V(t) + \frac{F(t)}{\lambda}\right] w, \quad t \in R \qquad (A.1)$$

with parameter $\lambda \in (0,\infty)$. Let $W(t,s,\lambda),\ t \geq s,\ s \in R$ be the evolution operator of system (A.1) on R^3 . We have

$$\begin{aligned} W(\omega,0,\lambda) &= \exp\left[\int_0^\infty \left(-V(t) + \frac{F(t)}{\lambda}\right) dt\right] \\ &= \exp\left[\int_0^\omega \begin{pmatrix} -g_1 & 0 & \frac{a_1(t)\Delta}{\lambda} \\ 0 & -g_2 & \frac{a_2(t)\Delta}{\lambda} \\ \frac{b_1\Sigma_1}{\lambda\Delta} & \frac{b_2\Sigma_2}{\lambda\Delta} & -\mu \end{pmatrix} dt\right], \forall\ \lambda > 0. \end{aligned}$$

It is easy to verify that system (2.1) satisfies assumptions (A1)–(A7) in Wang and Zhao [1]. Thus, we obtain the following two results, which will be used in our numerical computation of the basic reproduction ratio and the proof of our main result, respectively.

Lemma A.1. The following statements are valid:

(i) If $\rho(W(\omega,0,\lambda)) = 1$ has a positive solution λ_0, then λ_0 is an eigenvalue of L, and hence $R_0 > 0$.

(ii) If $R_0 > 0$, then $\lambda = R_0$ is the unique solution of $\rho(W(\omega,0,\lambda)) = 1$.

(iii) $R_0 = 0$ if and only if $r(W(\omega,0,\lambda)) < 1$ for all $\lambda > 0$.

Lemma A.2. The following statements are valid:

(i) $R_0 = 1$ if and only if $\rho(\Phi_{F-V}(\omega)) = 1$.

(ii) $R_0 > 1$ if and only if $\rho(\Phi_{F-V}(\omega)) > 1$.

(iii) $R_0 < 1$ if and only if $\rho(\Phi_{F-V}(\omega)) < 1$.

Thus, the disease-free solution E_0 is locally asymptotically stable if $R_0 < 1$, and unstable if $R_0 > 1$.

Lemma A.1(ii) shows that the basic reproduction rate is determined by parameter λ of $\rho(W(\omega, 0, \lambda)) = 1$. We can calculate the basic reproduction ratio R_0 using the numerical method.

Appendix B-Proof of the main result

Here we define $X := \{(P_1, P_2, \mathrm{y}) : 0 < P_1 \leq 1, 0 < P_2 \leq 1, 0 < \mathrm{y} \leq 1\}$ for system (2.1). It is easy to obtain the following theorem.

Theorem B.1. System (2.1) has a unique solution with the initial value

$$(P_1^0, P_2^0, \mathrm{y}^0) \in X := \{(P_1, P_2, \mathrm{y}) : 0 < P_1 \leq 1, 0 < P_2 \leq 1, 0 < \mathrm{y} \leq 1\},$$

and this compact set is positively invariant.

Define

$$X_0 = \{(P_1, P_2, \mathrm{y}) \in X : P_1 > 0, P_2 > 0, \mathrm{y} > 0\},\ \partial X_0 = X/X_0.$$

Let $H : X \to X$ be the Poincaré map associated with system (2.1), that is,

$$H(x^0) = u(\omega, x^0),\ \forall x^0 \in X,$$

where $u(t, x^0)$ is the unique solution of system (2.1) with $u(0, x^0) = x^0$. It is easy to see that

$$H^m(P_1^0, P_2^0, \mathrm{y}^0) = u(m\omega, (P_1^0, P_2^0, \mathrm{y}^0)),\quad \forall m \in Z_+,$$

where Z_+ is the set of all non-negative integers. Next, we establish the following lemma which will be useful in the proof of our main result.

Lemma B.1. If the basic reproduction ratio $R_0 > 1$, then there exists a $\sigma^* > 0$, such that for any $(P_1^0, P_2^0, \mathrm{y}^0) \in X_0$ with

$$\|(P_1^0, P_2^0, \mathrm{y}^0) - E_0\| \leq \sigma^*,$$

we have

$$\limsup d(H^m(P_1^0, P_2^0, \mathrm{y}^0), E_0) \geq \sigma^*. \qquad (B.1)$$

Proof. Since $R_0 > 1$, Lemma $A.2$ implies $\rho(\Phi_{F-V}(\omega)) > 1$. It follows that $\rho(\Phi_{F-V-M_\sigma}(\omega)) > 1$ holds for sufficiently small $\sigma > 0$, where

$$M_\sigma = \begin{pmatrix} 0 & 0 & a_1(t)\Delta\sigma \\ 0 & 0 & a_2(t)\Delta\sigma \\ b_1(t)\Sigma_1\sigma/\Delta & b_2(t)\Sigma_2\sigma/\Delta & 0 \end{pmatrix}.$$

By the continuity of the solutions with respect to the initial values, there exists a $\sigma^* > 0$ such that for all $(P_1^0, P_2^0, \mathrm{y}^0) \in X_0$ with $\|(P_1^0, P_2^0, \mathrm{y}^0) - E_0\| \le \sigma^*$, there holds $\|u(t, (P_1^0, P_2^0, \mathrm{y}^0)) - u(t, E_0)\| \le \sigma$, for all $t \in [0, \omega]$. Next, we claim that $\limsup_{m\to\infty} d(H^m(P_1^0, P_2^0, \mathrm{y}^0), E_0) \ge \sigma^*$. Assume, by contradiction, that $(B.1)$ does not hold. Then we have

$$\limsup_{m\to\infty} d(H^m(P_1^0, P_2^0, \mathrm{y}^0), E_0) < \sigma^*$$

for some $(P_1^0, P_2^0, \mathrm{y}^0) \in X_0$. Without loss of generality, we assume that $d(H^m(P_1^0, P_2^0, \mathrm{y}^0), E_0) < \sigma^*$, for all $m \ge 0$. It follows that $\|u(t, H^m(P_1^0, P_2^0, \mathrm{y}^0)) - u(t, E_0)\| = \|u(t, H^m(P_1^0, P_2^0, \mathrm{y}^0))\| < \sigma, \forall m \ge 0, \forall t \in [0, \omega]$. For any $t \ge 0$, let $t = m\omega + t'$, where $t' \in [0, \omega)$, and m is the largest integer less than or equal to t/m. Therefore, we have

$$\|u(t, (P_1^0, P_2^0, \mathrm{y}^0)) - u(t, E_0)\| = \|u(t', H^m(P_1^0, P_2^0, \mathrm{y}^0))\| < \sigma, \quad \text{for all } t \ge 0.$$

Note that $(P_1(t), P_2(t), \mathrm{y}(t)) = u(t, (P_1^0, P_2^0, \mathrm{y}^0))$. It then follows that $P_1(t) < \sigma, P_2(t) < \sigma, \mathrm{y}(t) < \sigma$, for all $t \ge 0$. From system (2.1), we get

$$\begin{cases} \dfrac{dP_1}{dt} \ge a_1(t)\Delta \mathrm{y}(1-\sigma) - gP_1, \\ \dfrac{dP_2}{dt} \ge a_2(t)\Delta \mathrm{y}(1-\sigma) - gP_2, \\ \dfrac{dy}{dt} \ge \left(\dfrac{b_1(t)\Sigma_1 P_1 + b_2(t)\Sigma_2 P_2}{\Delta}\right)(1-\sigma) - \mu \mathrm{y}. \end{cases} \qquad (B.2)$$

We then consider the following system

$$\begin{cases} \dfrac{d\hat{P}_1}{dt} = a_1(t)\Delta \hat{\mathrm{y}}(1-\sigma) - g\hat{P}_1, \\ \dfrac{d\hat{P}_2}{dt} = a_2(t)\Delta \hat{\mathrm{y}}(1-\sigma) - g\hat{P}_2, \\ \dfrac{d\hat{y}}{dt} = \left(\dfrac{b_1(t)\Sigma_1 \hat{P}_1 + b_2(t)\Sigma_2 \hat{P}_2}{\Delta}\right)(1-\sigma) - \mu \hat{\mathrm{y}}. \end{cases} \qquad (B.3)$$

By Zhang and Zhao ([3], Lemma 2.1), we know that there exists a positive, ω-period function $(\bar{P}_1(t), \bar{P}_2(t), \bar{\mathrm{y}}(t))^T$ such that $(\hat{P}_1(t), \hat{P}_2(t), \hat{\mathrm{y}}(t))^T = e^{\xi t}(\bar{P}_1(t), \bar{P}_2(t), \bar{\mathrm{y}}(t))^T$ is a solution of system $(B.3)$, where $\xi = \dfrac{1}{\omega} \ln \rho(\Phi_{F-V-M_\sigma}(\omega))$. It follows from $\rho(\Phi_{F-V-M_\sigma}(\omega)) > 1$ that ξ is a positive constant. Let $t = n\omega$, here $n \in Z_+$. We have

$$(\hat{P}_1(n\omega), \hat{P}_2(n\omega), \hat{\mathrm{y}}(n\omega))^T = e^{\xi n\omega}(\bar{P}_1(n\omega), \bar{P}_2(n\omega), \bar{\mathrm{y}}(n\omega))^T \to (\infty, \infty, \infty)^T \text{ as } n \to \infty.$$

For any nonnegative initial condition $(P_1(0), P_2(0), \mathrm{y}(0))^T$ of system $(B.2)$, there exists a sufficiently small $m^* > 0$ such that $(P_1(0), P_2(0), \mathrm{y}(0))^T \geq m^*(\bar{P_1}(0), \bar{P_2}(0), \bar{\mathrm{y}}(0))^T$. By the comparison principle, we have $(P_1(t), P_2(t), \mathrm{y}(t))^T \geq m^*(\hat{P_1}(t), \hat{P_2}(t), \hat{\mathrm{y}}(t))^T$, for all $t > 0$. Thus, we obtain $P_1(n\omega) \to \infty$, $P_2(n\omega) \to \infty$ and $\mathrm{y}(n\omega) \to \infty$, as $n \to \infty$, a contradiction. This completes the proof.

Based on the above work, we give the main result as mentioned in Materials and Methods, which may be written in the form of following theorem.

Theorem B.2. If the basic reproduction ratio $R_0 < 1$, then the unique disease-free solution $E_0 = (0,0,0)$ is globally asymptotically stable. Whereas if the basic reproduction ratio $R_0 > 1$, then there exists a constant $\delta > 0$ such that any solution $(P_1(t), P_2(t), \mathrm{y}(t))$ of system (2.1) with initial value $(P_1(0), P_2(0), \mathrm{y}(0)) \in X_0$ satisfies

$$\liminf_{t\to\infty} P_1(t) \geq \delta, \ \liminf_{t\to\infty} P_2(t) \geq \delta, \ \text{and} \ \liminf_{t\to\infty} \mathrm{y}(t) \geq \delta.$$

Proof: In the case $R_0 < 1$, Lemma A.2 implies that the disease-free periodic state E_0 is locally asymptotically stable. It is sufficient to prove that E_0 is globally attractive if $R_0 < 1$. From system (2.1), we have

$$\begin{cases} \dfrac{dP_1}{dt} \leq a_1(t)\triangle \mathrm{y} - gP_1, \\ \dfrac{dP_2}{dt} \leq a_2(t)\triangle \mathrm{y} - gP_2, \\ \dfrac{dy}{dt} \leq \dfrac{b_1(t)\Sigma_1 P_1 + b_2(t)\Sigma_2 P_2}{\triangle} - \mu \mathrm{y}. \end{cases} \tag{B.4}$$

Consider the following comparison system

$$\frac{dh(t)}{dt} = (F(t) - V(t))h(t). \tag{B.5}$$

Applying Lemma A.2, we know that $R_0 < 1$ if and only if $\rho(\Phi_{F-V}(\omega)) < 1$. By Zhang and Zhao ([3], Lemma 2.1), it follows that there exists a positive, ω-period function $\bar{h}(t)$ such that $h(t) = e^{\theta t}\bar{h}(t)$ is a solution of system (B.5), where $\theta = \dfrac{1}{\omega}\ln \rho(\Phi_{F-V}(\omega))$. Since $\rho(\Phi_{F-V}(\omega)) < 1$, then $\theta < 0$. Therefore, we have $h(t) \to 0$ as $t \to +\infty$. This implies that the zero solution of system (B.5) is globally asymptotically stable. For any nonnegative initial value $(P_1(0), P_2(0), \mathrm{y}(0))^T$ of system (B.4), there is a sufficiently large $M^* > 0$ such that $(P_1(0), P_2(0), \mathrm{y}(0))^T \leq M^*\bar{h}(0)$ holds. Applying the comparison principle [4], we have $(P_1(t), P_2(t), \mathrm{y}(t))^T \leq M^* h(t)$, for all $t > 0$, where $M^* h(t)$ is also the solution of system (B.5). Therefore, we get $P_2(t) \to 0$, $P_2(t) \to 0$ and $\mathrm{y}(t) \to 0$ as $t \to +\infty$. We finish the proof of the first part.

By Theorem B.1, the discrete-time system $\{H^m\}_{m\geq 0}$ admits a global attractor in X. Now we prove that $\{H^m\}_{m\geq 0}$ is uniformly persistent with respect to$(X_0, \partial X_0)$. Clearly, there is exactly one fixed point $E_0 = (0,0,0)$ of H in ∂X_0. Lemma B.1 implies that $E_0 = (0,0,0)$ is an isolated invariant set in X and $W^S(E_0)\bigcap X_0 = \varnothing$. By Zhao ([5], Theorem 1.3.1), it follows that H is uniformly persistent with respect to $(X_0, \partial X_0)$. By Zhao ([5], Theorem 3.1.1), the solutions of system (2.1) are uniformly persistent with respect to $(X_0, \partial X_0)$, that is, there exists a constant $\delta > 0$ such that any solution $(P_1(t), P_2(t), \mathrm{y}(t))$ of system (2.1) with initial value $(P_1(0), P_2(0), \mathrm{y}(0)) \in X_0$ satisfies

$$\liminf_{t\to\infty} P_1(t) \geq \delta, \ \liminf_{t\to\infty} P_2(t) \geq \delta, \ \text{and} \ \liminf_{t\to\infty} \mathrm{y}(t) \geq \delta.$$

References

[1] Wang WD, Zhao XQ: **Threshold dynamics for compartmental epidemic models in periodic environments.** *J Dyn Diff Equat* 2008, **20:** 699-717.

[2] P., Watmough, J.: **Reproduction numbers and sub-threshold endemic equilibria for compartmental models of disease transmission.** *Math Biosci* 2002, **180:** 29-48.

[3] Zhang F, Zhao XQ: **A periodic epidemic model in a patchy environment.** *J Math Anal Appl* 2007, **325:** 496-516.

[4] Smith HL, Walman P: *The Theory of the Chemostat.* Cambridge: Cambridge University Press; 1995.

[5] Zhao XQ: *Dynamical Systems in Population Biology.* New York: Springer; 2003.
